# Toward a Hybrid Theory of How to Allocate Health-related Resources

**DOI:** 10.1093/jmp/jhad022

**Published:** 2023-06-06

**Authors:** Anders Herlitz

**Affiliations:** Institute for Futures Studies, Stockholm, Sweden, Lund University, Lund, Sweden; University of Gothenburg, Gothenburg, Sweden

**Keywords:** commitments, comparability, healthcare rationing, pluralism, priority setting

## Abstract

How should scarce health-related resources be allocated? This paper argues that values that apply to these decisions fail to always fully determine what we should do. Health maximization and allocation-according-to-need are suggested as two values that should be part of a general theory of how to allocate health-related resources. The “small improvement argument” is used to argue that it is implausible that one alternative is always better, worse, or equal to another alternative with respect to these values. Approaches that rely on these values are thus incomplete. To deal with this, it is suggested that we ought to use incomplete theories in a two-step process. Such a process first discards ineligible alternatives, and, second, uses reasons grounded in collective commitments to identify a unique, best alternative in the remaining set.

## I. INTRODUCTION

Scarcity of health-related resources constitutes a major challenge both for contemporary societies and for contemporary political philosophy (Bognar and Hirose, 2014). All societies, rich and poor, must ration health-related resources, and it is reasonable to predict that complications in relation to this will only increase (Robinson et al., 2011). How, then, *should* societies distribute their scarce health-related resources? Which criteria should be used to justify such distributions? Most philosophers who write on this issue accept some kind of pluralism, that several values—or several value dimensions—are relevant when different distributions are evaluated (e.g., Segall, 2009; Beauchamp and Childress, 2012; Eyal et al., 2013; Nielsen, 2013; Esposito and Hassoun, 2017). Such pluralism actualizes the challenge of determining how to weigh and/or combine the different values in order to form an overall judgment about what distribution is best. In this paper, I argue that the values that apply to evaluations of different distributions fail to always determine a best distribution, and I outline what this implies for how to make justified choices in this area.

The argument is straightforward. In the second section, I suggest that no monistic approach to this issue is satisfactory, and that we thus should accept a pluralistic approach. I furthermore suggest that the values *health maximization* and *allocation-according-to-need* should be part of such a pluralistic approach. In the third section, I argue that, whichever pluralistic approach we embrace, we ought to accept that it is *incomplete* in the sense that its application will not generate a determinate answer to which of the alternatives is best in all situations. In the fourth section, I examine what incomplete decision criteria can do for us. I argue that we should use incomplete principles only to determine which alternatives are ineligible with respect to the values. In the fifth and final section, I invoke contemporary philosophical research on practical reason that suggests that not all reasons are given, but that some reasons, through commitments, are *created* reasons, and that such reasons enable comparisons of alternatives, the relation between which is underdetermined. I suggest that a similar approach can be taken to decisions that concern allocation of scarce health-related resources. In that vein, I conclude that we need a two-step process in order to deal with these issues: *given* reasons provided by the plural values that we ought to embrace are superior to *created* reasons, and they establish which alternatives are ineligible, whereafter we must invoke reasons that are created through collective commitments in order to select which of the eligible alternatives to choose. Since these collective commitments are today absent, societies at large ought to address what they want to be in order to be able to provide reasons that can generate determinate evaluations of alternative health resource allocations.

## II. VALUES AND HEALTH POLICY

No *one* value can reliably be used to evaluate alternative distributions of health-related resources in a plausible way. Although this is an assumption I will make in this paper, I introduce in this section two values that seem defensible and unavoidable when we search for guidance about how to allocate health-related resources. The values are *health maximization* and *allocation-according-to-need*. The purpose of introducing two values is to illustrate the complications that arise once one accepts pluralism. Pluralism, it should be noted, is not a controversial view (Robinson et al., 2011). It is accepted by many who work within the consequentialist tradition that maximization of a single good, such as health, is not the only thing that matters, since, for instance, the *fairness* of distributions also matters. Many consequentialists accept that inequalities are undesirable and that an outcome with less inequalities in health is in some respect better (Eyal et al., 2013). This can take different expressions. For example, some consequentialists are drawn toward prioritarianism and hold that one ought to evaluate distributions of health-related resources by summing up the amount of health benefits produced but ascribe extra weight to benefits to the worse off (Ottersen, 2013). However, pluralism is also recognized by researchers who conceptualize consequentialism as maximization of health and think of fairness considerations as separate deontological criteria (Beauchamp and Childress, 2012). Such researchers, for instance, might hold that beneficence is good, so that it is commendable to promote good health, but also accept that certain requirements of justice put constraints on health promotion, so that health maximization is not a generally uniquely valid normative principle.

As one accepts the need for pluralism, regardless of whether one thinks of this as a combination of consequentialist and deontological ethics or as a kind of consequentialism (and regardless of what set of substantive values one accepts), the issue of whether we can develop a *complete* theory of how the plural values should guide our behavior arises: is the pluralism such that we, in every instance (in which the different values might conflict), can establish what we ought to do in light of those values? In the next section, I use the values of health maximization and allocation-according-to-need to argue that the answer is no.

An approach with great appeal that is often used in health economics proposes *maximization of health* as the standard by which we can evaluate rationing alternatives. The simple idea behind this approach is similar to utilitarianism: there is a single value, *aggregate health*, that matters, and we ought to distribute resources so that this value is maximized. In order to maximize health, one needs some way of measuring health outcomes. The notion *QALY* (*Quality-Adjusted Life Year*) has been suggested as such a measurement that can be used to calculate aggregate health outcomes to be achieved by various allocation alternatives and, therefore, can be used as a standard by which allocation alternatives can be compared (Sassi, 2006; Bognar and Hirose, 2014). QALYs are calculated by multiplying years that will be lived with a certain *quality of health* expressed on a scale between 0 and 1, where 1 is perfect health. One year of life of an individual in perfect health amounts to 1 QALY (1 year × 1 health quality = 1). Half a year lived at perfect health amounts to 0.5 QALY (0.5 × 1 = 0.5). A year of life at significantly reduced quality of health (say, quality of health = 0.5) amounts to 0.5 QALY (1 × 0.5 = 0.5). And so on. In order to evaluate allocation outcomes, the total value of aggregate health can be measured in terms of QALYs so that one can see which alternative produces the most QALYs. QALY is thus aggregative. In its simplest form, QALY is purely additive, so it can be used to express the value of health outcomes of very large-scale interventions: e.g., 35,000 years at perfect health (35,000 × 1), 12,000 years at quality of health level 0.9 (12,000 × 0.9), and 3,000 years at quality of health level 0.4 (3,000 × 0.4) amount to a combined total of 47,000 QALYs. Putting aside the practical issues of how to actually establish the quality of health level of individuals, it is easy to see how QALYs can be used as a standard by which one can compare and evaluate alternative allocations (Bognar and Hirose, 2014). Although controversial and surrounded by both theoretical and practical challenges (cf. Hausman, 2015), in what follows, I focus on QALY when I discuss health maximization because it enables a desirable clarity.

To rely solely on unqualified aggregate QALY maximization, or any other aggregate health maximization, implies deeply counterintuitive conclusions, since no importance is assigned to how much different individuals *need* health resources, or to the *fairness* of the distribution. When calculating QALYs, no importance at all is assigned to the differences in individual need for health resources that different persons have. A policy that cures 10 individuals of cancer so that they can live at perfect health for a total of 300 years (30 years each) scores lower (300 QALYs) than, and is thus considered inferior to, a policy that provides myopia training devices to 11,000 individuals who run the risk of developing nearsightedness so that they can live at a health level that is 0.01 higher for a total of 33,000 years (330 QALYs). QALY maximization is, in other words, insensitive to the different importance of health needs. QALY is also insensitive to the distribution of health resources and benefits: In a situation where one needs to choose between implementing a health policy that only benefits people in the capital region of a country and a health policy that provides health resources throughout the whole country, the QALY standard will deem it correct to allocate all resources to the capital region, if that maximizes QALYs.

For these reasons, we need to abandon the idea that maximization of aggregate health outcomes can function as *the exclusive* standard when alternative allocations of health-related resources are evaluated. Since similar problems (familiar, of course, from debates on utilitarianism) arise, and are easy to spell out, for all distributive principles that propose the maximization of a certain value, we need to abandon the idea that any standard that relies on the maximization of a certain value can function as the conclusive standard in healthcare rationing.

The discussion above shows the relevance of a different value that is often suggested in relation to health care: health-related resources ought to be allocated so that they satisfy the greatest health needs, *allocation-according-to-need* (Robinson et al., 2011; Juth, 2015; Herlitz and Horan, 2016; Herlitz, 2017). This value enters the debate in somewhat different shapes, but the core idea is that a distributive principle that we ought to embrace ranks alternative allocations based on how much various recipients need the benefits that they gain. Needs are held to be greater, the larger the benefit someone can get, and the more serious the medical conditions are (Williams, 1962; Elster, 1995; Herlitz and Horan, 2016; Herlitz, 2017). A patient with a lethal—but curable—disease has greater health needs than a patient with a disturbing skin condition.

Allocation-according-to-need approaches can deal with some of the problems that health maximization approaches face. Applying allocation-according-to-need to the examples above generates results that are more intuitively acceptable. According to allocation-according-to-need, we should prioritize cancer patients since they clearly have a more serious medical condition, which means that benefits to them count for more so that the value of benefiting cancer patients is greater than the value of handing out myopia training devices, even if the total amount of health benefits is smaller. We can also see how discriminatory policies can be counteracted by allocation-according-to-need, since discriminated groups often are worse off and thereby ascribed greater importance and priority.

## III. INCOMPLETE PLURALISM

Health-related resources ought to, prima facie, be allocated so that they *maximize health*. Furthermore, health resources ought to, prima facie, be allocated according to *allocation-according-to-need* so that the individuals in the population with the greatest need for health-related resources are prioritized. This is the intuition that is captured and expressed by need-based approaches, and we see that it matters when we look at allocations that fail to take the different importance of individuals’ health needs into account.

However, the most efficient allocation in terms of health maximization is not always the allocation that secures that those needing resources the most will receive them. In order to deal with the value conflicts that arise, one needs some way of weighing, or of ordering, these values.

In order to address this issue, let us measure health maximization with QALY and measure allocation-according-to-need with the “Neglected Weightier Needs Score.” QALY was introduced above. Although this measure has significant shortcomings, it adequately enough captures the core idea and intuition behind health maximization for our purposes. We ought to allocate health resources so that we create the best possible aggregate health outcome. In other words, for the sake of this argument, prima facie, the more QALYs we can generate the better.

Here I introduce the *Neglected Weightier Needs Score*, henceforth *NWNS*, as a measure for how well allocation-according-to-need is satisfied by a specific resource allocation. NWNS measures the weighted distance between a health outcome and perfect expected lifetime health, which is to say that it measures severity-weighted ill-health in a population.

To measure severity-weighted ill-health, we need to measure aggregate health shortfalls in a population. The mathematics involved in representing and measuring aggregated shortfalls is more complicated than what philosophers are typically used to, but a simple approach from poverty research can be borrowed for this context: the so-called Foster-Greer-Thorbecke indices (cf. Foster, Greer, and Thorbecke, 1985, 2010; Alkire et al., 2015; Herlitz and Horan, 2016 and 2017). These indices measure weighted, aggregated gap closures in the following way (Foster, Greer, and Thorbecke, 1985, 2010):


$$\begin{array}{l} \frac{1}{N}\underset{i=1}{\overset{H}{\sum }}\;(\frac{z-y_{i}}{z})\alpha \end{array}$$

For our purposes, *z* can be interpreted as a health benchmark expressed in lifetime QALYs (i.e., perfect expected lifetime health), *y*_*i*_ is expected lifetime health of individual *i*, *N* is the number of individuals in the whole population, and *H* is the number of individuals below the benchmark (people who are expected to suffer from ill-health). Finally, *α* is the weighting scheme. The larger the *α*, the more weight is placed on the worse off individuals. This function provides a relatively simple way of measuring weighted aggregated shortfalls, and it can also serve as an illustration of how we can measure unsatisfied severity-weighted health needs.

By looking at normalized health shortfalls in this way, we can construe an indicator that takes a value between 0 and 1 and tells us how good an outcome is in terms of allocation-according-to-need. The closer its value is to 1, the better the outcome. The closer to 1, the smaller the disvalue according to allocation-according-to-need. What specific weighting scheme to use depends on how much weight one gives to severe conditions, but whichever weighting scheme one uses, it can be used as an indicator of gap closures so that the indicator produces results that correspond to the specific type of allocation-according-to-need view promoted.

Consider now the possibility that a decision-maker might be forced to choose between two alternative allocations of health resources, X and Y, such that expected QALY_X_ > expected QALY_Y_ and expected NWNS_X_* *< expected NWNS_Y_. Consider, for example, the following outcomes of alternative health resources allocations:

*Allocation A*: Expected QALYs of implementation: 30 000; expected NWNS: 0.5.

*Allocation B*: Expected QALYs of implementation: 20 000; expected NWNS: 0.7.

Which alternative is, in choices like this, the better one? Aggregate health is maximized if we choose Allocation A, but if we choose Allocation B, we meet severity-weighted needs to a higher extent.

One response to difficult choices like these would be to claim that, unless one alternative is better than the other, the alternatives are equally good. This follows if we recognize that no alternative is better than the other and stick firmly to the so-called *trichotomy thesis* (Chang, 1997, 2002; Rabinowicz, 2008), the idea that the set of possible positive relations between two alternatives, X and Y, is exhausted by the trichotomy: {*X is better than Y*, *Y is better than X*, *X and Y are equally good*}. According to the trichotomy thesis, if A is not better than B, and B is not better than A, it follows that they must be equally good.

We should be careful to draw that conclusion, however. The trichotomy thesis does not hold in these cases. In order to see why we should reject the trichotomy thesis, assume that it is false that either of the above alternatives is better than the other and let us ask: does it make sense to rank the alternatives in a *categorically* different way if we make slight adjustments to the alternatives (cf. Chang, 2002; Andersson and Herlitz, 2022a)? Consider the following:

*Allocation A’*: Expected QALYs of implementation: 30 001; expected NWNS: 0.5.

*Allocation B’*: Expected QALYs of implementation: 20 000; expected NWNS: 0.6999.

It is hard to see how these minimal adjustments to the allocations would imply a categorically different positive relation between the alternatives and facilitate the decision. It is, of course, obvious that A’ is better than A, and that B’ is better than B, but it does not strike me as clear that A’ is better than B, or B’ better than A. Yet, if the alternatives A and B were truly equally good, then it would follow that even the slightest improvement of one alternative would break the tie. If A is equally as good as B and A’ better than A, then A’ is better than B; and if A is equally as good as B and B’ better than B, then B’ is better than A.

In order to stress this point further, let me introduce a more concrete thought experiment. Imagine that you have $1 billion to distribute across different health-promoting institutions in a society. At hand, you also have the most refined weighted maximization standard imaginable that combines the values health maximization and allocation-according-to-need. Call the standard *PERFECT*. As it happens, when you apply PERFECT to your situation, it identifies *two* maximal allocations, P and Q, neither of which is worse than the other. P is a very good allocation alternative. It distributes the resources so that very high numbers of QALYs will be generated and a large part of the most pressing needs will be satisfied. Q is also a very good allocation, but it is quite different. Q will distribute the resources so that almost all the most pressing needs will be satisfied, and will do better than P in terms of this, but it will generate fewer QALYs.

It is, in this situation, peculiar to hold that P and Q are equally good. In order to see this, consider what happens if you realize that you made a very small miscalculation when you estimated the value of P. In fact, one of the benefits included in P but not Q was slightly cheaper than you thought, so you must revise your estimations and include an additional very small benefit in P: you can give this to Dennis, who has some risk of developing nearsightedness, if you choose P, so that you can in fact choose allocation P+ (i.e., all the good aspects included in P, plus the health benefits that can be generated by giving a myopia training device to Dennis). If P and Q truly were equally good, then you must conclude that this change of the estimations makes it an easy choice. P+ is categorically better than both P and Q and must be chosen. But this seems wrong. The choice between P and Q is the choice between what to value the highest in this instance, satisfying pressing needs or maximizing health?

The conclusion to draw from this discussion is rather that we should consider the relation between Allocation A and Allocation B, or, more abstractly, between some outcomes, as not determined. We could interpret this as a merely epistemological problem (cf. Hirose, 2015). Perhaps an omniscient being could establish determinate positive relations between all outcomes that score differently in terms of QALYs and NWNS in accordance with the trichotomy thesis, so that one alternative always is better than, worse than, or equally good as another alternative in terms of QALY and NWNS. If that were so, then perhaps we would be able to solve these dilemmas by gathering more information. This strikes me as implausible.

QALY and NWNS express values that contribute to the overall goodness of an outcome in fundamentally different ways. Occasionally, there is no large difference in the degree to which they exemplify their way of contributing to the overall goodness of an outcome; and contributing to the overall goodness of an outcome in one of the ways is not categorically superior to meeting it in the other way. Within the quite limited research on the conditions under which the trichotomy thesis fails, this is often suggested to be the underlying explanation of the failure (Raz, 1986; Anderson, 1993). Sometimes this is called “incommensurability,” but a more careful conclusion is to only dismiss the validity of the trichotomy thesis (cf. Herlitz, 2019, 2020). It is hard to see how *more information* could establish how values relate to each other under the described conditions. A better explanation is, thus, to abandon the trichotomy thesis and to accept that there are other positive relations that alternative allocations can hold to each other. We could accept that Allocation A and Allocation B, or, more abstractly, some allocations, are *on a par* or *roughly equal* (Parfit, 1984; Chang, 2002). However, when we turn to the practice of how to allocate health-related resources, it is of limited importance whether we consider this an epistemological problem or an axiological fact, as long as we remain unable to settle the issue (cf. Andersson and Herlitz, 2022b).

However one chooses to operationalize the values captured by health maximization and allocation-according-to-need, there is a non-empty set of pairs of allocations, the relation between which is not determined, so that neither alternative is better than the other; but they are not equally good, either. Otherwise put, the pluralism that we need to embrace in order to accommodate for our intuitions about how to allocate health-related resources and the given values that we need to adhere to—that both health maximization and allocation-according-to-need matter—cannot be complete in the sense that its application in every situation generates a conclusive answer to which alternative is all-things-considered best.

This is, then, also the reason why we should abandon attempts to develop *complete* weighted maximization strategies. However one designs such strategies, there will be an allocation {QALY_x_; NWNS_x_} and an allocation {QALY_y_; NWNS_y_} that are evaluated as equally good, according to the complete weighted maximization strategy. Now whichever {QALY_x_; NWNS_x_} and {QALY_y_; NWNS_y_} are, it is possible to illustrate how absurd it is to consider them to be equally good by pointing out that diminishingly small adjustments would make a categorical difference according to the approach. Will the addition of a myopia training device to one alternative categorically change the relation between them? It is, in other words, impossible to establish a decision standard, based on the given values of health maximization and allocation-according-to-need, that can be applied to, and generate a conclusive answer to, the question of which alternative is the best in situations in which a certain amount of health-related resources must be distributed across a diverse population with diverse needs.

A general approach to how to allocate health-related resources must be more comprehensive than the one discussed above. We need to also recognize that equality matters, that some resources should be allocated based on willingness to pay, and perhaps also other normative considerations. However, the fact that the discussion above is highly simplified only strengthens the argument for incompleteness. Arguments similar to the one above can be generated for conflicts between allocation-according-to-need and equality, between equality and health maximization, and for situations where all three of these values conflict. And, I contend, the same phenomenon arises for certain pairwise comparisons of allocation alternatives, whichever values we use in order to guide allocation decisions, insofar as we do not adhere to a monistic approach and insofar as the values contribute to the overall good in fundamentally different ways.

## IV. DEALING WITH INCOMPLETENESS

In the previous two sections, I suggested that we need to invoke at least two values, health maximization and allocation-according-to-need, when we attempt to establish justified strategies for allocating scarce health-related resources on the macro-level, and I argued that no theory that incorporates these two values can be complete in the sense that it provides conclusive answers to which alternative is the best all-things-considered in all pairwise comparisons of allocation alternatives. In this and the following section, I address how we can deal with the fact that it is impossible to establish a justified, complete decision strategy for how to allocate health-related resources. In this section, I first address the objection that hard cases make bad law and show that this otherwise sound maxim does not apply to this context. Once this objection is out of the way, I address what incomplete decision standards can contribute to a theory of how to allocate scarce health-related resources. I claim that these can function as the first step to exclude ineligible allocation alternatives in a more complex process. In the next section, I sketch how societal, collective commitments can complement the incomplete decision strategies.

Let me start with addressing a worry that some might have with the argument in the previous section. It is sometimes said that hard cases make bad law, and the argument in the previous section largely relied on pointing out shortcomings with certain decision strategies in the face of hard cases. Maybe these cases are so rare that it is better not to focus on them when we develop a general approach.

This objection is not convincing in the context of this paper. Allocating scarce health-related resources is a maximization venture where we can expect clusters of hard cases to gather, the closer we get to the best alternative. In the discussion in the previous section, I simplified matters so that there are only two/four alternative outcomes. Macro-level resource allocation is rarely, if ever, this simple. Rather, decision-makers face an indefinite number of alternative allocations of the resources available. There is an indefinite number of alternative recipients of health-related resources, and the pile of resources can be divided up in a vast number of different ways. A large number of the possible allocations can easily be dismissed as suboptimal, but, when this is done, we still have a large number of very different alternatives to choose between. The difficult, and interesting, issues that arise when the obviously suboptimal alternatives have been put aside and we need to select between alternatives that cannot be easily compared are ubiquitous, and hard cases are quite likely more common than easy cases. Hard cases are exactly what we should focus on when we discuss these issues.

The incompleteness conclusion above might appear disappointing, but there are in fact significant upshots of accepting that we ought to settle for incomplete theories of how to allocate health-related resources. First, it means that we can focus on the issue that we inevitably are bound to face, instead of wasting time searching for a philosophers’ stone that could provide us with answers to all problems but that does not exist. Second, and perhaps more important, it helps us avoid the quite serious risks connected with applications of *assumed-to-be*-complete principles. All allocation alternatives can, in a practical sense, always be compared and uniquely ranked *somehow* (cf. d’Agostino, 2003). Even when it is not determined which alternative is the better one, we can, of course, apply QALY maximization, NWNS, PERFECT or any other decision standard in order to establish *some* ranking of the alternatives (cf. Herlitz, 2016). The consequences of applying an unjustified standard are, however, troublesome. Repeated application of an unjustified decision strategy leads to institutionalization of unjust distributions of resources. If we systematically apply an assumed-to-be-complete decision standard to situations where it is, in fact, not determined which alternative is the better one, certain groups might be systematically discriminated against (cf. Herlitz, 2023). We might not know *which group*, but we know the decisions made are not satisfactorily justified, which means that we can expect that some injustice is likely committed by the application of complete decision standards in allocation decisions. In more concrete terms, some socio-economic group might be favored, certain health needs might be undervalued, some ethnic groups might be discriminated against, adherents to certain religions might be discriminated against, and/or gender inequalities might be institutionalized. By accepting that decision standards need to be incomplete, we might avoid these outcomes, whatever their exact nature may be. Abandoning the ideal of complete decision standards is thus a way to avoid potential injustices.

How to deal with incomplete theories is an issue that is receiving increasing attention in various research areas due to increased recognition of the problem. Within axiology, a growing amount of research points toward the conclusion that the trichotomy thesis fails, and that there are more positive relations between values than *better than*, *worse than*, and *equal to* (Chang, 2002; Rabinowicz, 2008; Carlson, 2010; Herlitz, 2019). Since Derek Parfit’s (1984) seminal introduction of the *mere addition paradox*, a large literature has addressed how to interpret what Larry Temkin (2012) calls *spectrum arguments*, which point toward the need for incomplete theories (Rachels, 1998; Persson, 2006; Herlitz, 2018). Certain insightful economists draw the same conclusion as they recognize that it might in fact be impossible to provide a complete order for certain social goods such as equalities (Sen, 1997, 2017).

Generally, there seem to be three different types of responses to the impossibility to provide a complete decision strategy. Sometimes, it is suggested that we apply a known-to-be-flawed theory to the problem. Amartya Sen (1997), for example, took part in the development of the *Human Development Index*, although he seems to have known this to be an imperfect measure of human development). In certain contexts, there can be good reasons to apply imperfect methods. Ruth Chang (2013) has recently, relying on a Korsgaardian Kantianism, suggested that the breakdown of the trichotomy thesis reveals that individuals can *create* reasons when reasons that are on a par fail to be conclusive (Korsgaard, 2009). Temkin (2012) and, long before him, Thomas Nagel (1979) suggest that we, instead, take an Aristotelian approach to the problem and invoke the importance of non-instrumental practical wisdom. None of these responses seem particularly promising when we deal with macro-level allocation decisions, and there is still significant work that needs to be done in this field. In the next section, I propose a re-interpretation of Chang’s proposal for this context. Before doing that, however, we must see what we can do with the incomplete decision strategies, because, although insufficient, they can provide significant assistance in the search for the just distribution of health-related resources.

What sort of condition could be used in order to use an incomplete principle to establish what alternatives are not eligible? The most straightforward way to use incomplete principles has been suggested by Amartya Sen. Sen (1997, 2017) suggests that when principles are incomplete, we ought to abandon optimization and instead look for alternatives that are *maximal*. An alternative is maximal if it is *not worse* than any alternatives. Thus, in the example above, although we might be unable to say that any of the outcomes A and B are best, we can say that both of them are maximal. When the choice set is expanded so that also A’ and B’ are available, we can say that although there is no optimal alternative, there are only two maximal alternatives, A’ and B’ (because A is worse than A’ and B is worse than B’). It is clear that this condition can be used in order to make incomplete principles partition choice sets into maximal and non-maximal alternatives. I suggest that we consider non-maximal alternatives ineligible.

Sen seems to believe that we can go further and also consider all maximal alternatives permissible. I believe that this is a mistake, for two reasons. First, when the relation between two outcomes is underdetermined, there is what Chang (2002) has called a “resolutional remainder.” We need some reason to resolve the problem, and it is a mistake to plump the decision and consider the alternatives equally permissible. Second, in social choice situations when the decision has a large impact on a big group of people (like when scarce health-related resources are allocated at the macro-level), decisions must be legitimized (cf. Herlitz and Sadek, 2021). That something is an optimal decision gives the decision some legitimacy. That something is maximal does not give the decision the same legitimacy.

In the next section, I suggest that we should select among maximal, but not optimal, alternatives in a certain way. Then I argue that the final step of the two-step process on which these decisions should be based consists of forming *commitments* to certain values.

## V. COMMITMENTS

In the literature on practical reason and comparability problems related to practical reasons, Chang (2013) has recently suggested that a way in which we can deal with parity is by invoking reasons that are created. Chang argues that there are three fundamentally different approaches to the issue of what grounds practical reasons, and she argues that we ought to accept a hybrid approach that combines some of the merits of each of these approaches. She calls this view “hybrid voluntarism.” The different approaches to what grounds a practical reason are familiar to moral philosophers. There are *externalists* who hold that reasons are grounded in facts “outside” of us, e.g., the fact that something is painful provides us with reasons to avoid it (e.g., Scanlon, 1998; Parfit, 2011). There are *internalists* who hold that reasons are grounded in facts “inside” us, e.g., the fact that I desire to avoid pain provides me with a reason to avoid things that cause pain (e.g., Williams, 1981; Smith, 1995). Additionally there are, Chang suggests, *voluntarists* who argue that reasons are grounded in our “willing” something, e.g., the fact that I will to live a life with minimal pain gives me a reason to avoid pain (e.g., Korsgaard, 2009).

Having identified these three approaches, Chang goes on to argue that the core and most interesting aspects of both the historic and the contemporary debates revolve around the issue of whether reasons are *given* or *of our own making*. Based on this distinction, Chang suggests that we ought to embrace a hybrid view where given reasons (best explained either with externalist or internalist accounts) provide us with reasons for how to act up to the point where these reasons are no longer conclusive, i.e., where it is underdetermined what you have most reason to do according to these reasons. Under such conditions, Chang claims, it does not suffice to say that anything goes, that we can just pick between equally permissible alternatives. Rather, we need to, through an act of willing, create reasons that guide us and ground a most reasonable choice: “given reasons operate as *metaphysical* constraints on voluntarist ones; we cannot bring voluntarist reasons into existence unless our given reasons fail *fully* to determine what to do” (Chang, 2013, 178). In other words, Chang presents a pluralism about the grounds of normative reasons: there are given reasons, and there are reasons that are grounded in individual acts of willing. In this pluralist model, given reasons take priority over created reasons; created reasons are only actualized, indeed only possible, when the given reasons fail fully to determine what to do.

Whether Chang is right or not when it comes to practical reasons is an issue we can here sidestep. In what follows, I instead argue that her suggestion has significant relevance for, and applicability to the field of, political philosophy and, in particular, for the issue of how to allocate scarce health-related resources. The structure of the problem that motivates Chang’s (1997, 2002) proposal, the failure of given reasons fully to determine what to do, is the same as the structure of the problem that we face when we search for a decision strategy when we allocate health-related resources. I have suggested that there are *given* reasons (or values) that we are normatively required to adhere to when we form allocation decisions. I above suggested that one of these reasons is to promote health maximization and that another is to promote allocation-according-to-need. However, as argued above, these reasons fail to fully determine how we should allocate scarce health-related resources. What Chang shows us is that we can deal with such incompleteness by invoking created reasons. This strategy is open to us also when we address political-philosophical issues such as allocation of health-related resources. However, the strategy needs to be modified.

When one addresses created reasons in relation to practical reasons, these reasons are suggested to be grounded in an individual act of willing (Korsgaard, 2009; Chang, 2013). Clearly, no single individual can, through an act of willing, create a valid reason that can establish how a society ought to allocate scarce health-related resources. That, quite simply, would be dictatorial. In this regard, Chang’s proposal and the idea of created reasons must be reinterpreted for social choice. Instead of an individual act of willing, I propose that a society can create reasons that can determine which alternative among the maximal but not optimal alternatives (i.e., within constraints posed by given reasons) is the better one with reference to a *collective act of willing*.

How are we to understand a collective act of willing? One interpretation would suggest something similar to Rousseau’s (1979)*volonté generale*. It is the aggregated will of the people, such as this exists perhaps unknown to the people, which can provide us with created reasons. This interpretation might seem natural in light of contemporary theory on voluntarist reasons that refers heavily to Kant, who in turn was greatly inspired by Rousseau (cf. Korsgaard, 1996, 2008, 2009). The Rousseauian interpretation is, however, for familiar reasons, a dangerous path to take. It is hard to learn what the *volonté generale* actually amounts to, and it provides a way for policy makers to impose policies supported by their own opinions on the public (cf. Popper, 1945; Berlin, 1969).

A more prudent approach is, instead, to suggest that collective acts of willing are collective acts of willing *knowingly* and *purposefully* expressed through legitimate liberal-democratic institutions such as elections. According to this interpretation, we can, as societies, create reasons through collective acts of willing where individuals knowingly and purposefully commit to specific values through legitimate liberal-democratic institutions (cf. Herlitz and Sadek, 2021). Such commitments can function as foundational ideals that establish the identity of society itself; they are commitments that set precedents for future decisions and that guide policy in hard cases. The reasons created in this manner will be valid reasons that apply to choice situations in which the given reasons fail fully to determine what we ought to do, and they provide legitimacy to the choices made in light of them. Within a just, democratic society, these reasons that are created by collective acts of willing inherit validity from the system in which they are created. If the institutional framework within which collective acts of willing are expressed is just, and if the process in which the commitments are made is just, then the commitments that are generated will be just. Societies can thus commit to certain values of the second order that can guide choice in hard cases. Perhaps a society in this way commits to solidarity with the weak, perhaps it commits to meritocratic ideals, or perhaps it commits to transparent technocracy. All of these ideals provide new ways of evaluating the not ineligible alternatives so that a unique, best choice can be identified.

The current absence of such commitments and of such created reasons reveals an identity crisis of contemporary societies. It is time we take this issue seriously. Electing representatives in very general elections is not knowingly and purposefully taking part in the collective act of willing a reason into existence, or collectively creating a commitment. For that, we need a public debate on the issue, and we need members of society to take part purposefully in such a process and to express their individual opinions on what such commitments should be (cf. Herlitz and Sadek, 2021). We need members of society to decide in what sort of society they hope to live. Absent such commitments, allocation of scarce health-related resources remain partly arbitrary, unjustified, discriminatory, and be a practice colored by the biases and prejudices of decision-makers (cf. Herlitz, 2023).

One could now, of course, suggest that we in fact ought to make a commitment to QALY maximization, or to a different modified QALY measure, in order to deal with allocation of health-related resources. However, we should acknowledge what such a commitment means. First, such a commitment would still be constrained by the given reasons, and it would thus differ from unqualified QALY maximization. The commitment would not be action-guiding when QALY maximization and NWNS fully determine a best alternative, and it would not sanction a decision that is not maximal with respect to QALY maximization and NWNS. Given reasons have priority in this model, and forming a collective commitment to QALY maximization only provide a reason that can guide choice among maximal but not optimal alternatives. As such, a collective commitment to QALY maximization differs from mere implementation of QALY maximization.

Second, in light of the discussion above, such a commitment cannot be a commitment merely to the values that are captured by the measures. Rather, it has to be a commitment to a technocratic decision strategy that can be invoked also in hard cases. As such, the commitment cannot be grounded in the values themselves, but must rather be grounded in values such as simplicity, transparency, and usability. This appears to me an unattractive, but, I submit, still feasible, path to take.

To summarize, I suggest that the way in which we ought to allocate scarce health-related resources is through a two-step process. We ought to (1) adhere to the given reasons that are available to us and use these to identify which the maximal alternatives are. In order to do this, we can invoke QALY maximization, NWNS, PERFECT, or other incomplete weighted maximization strategies. Frequently, no alternative will be optimal, for reasons outlined in Section II above. We recurrently find ourselves with a set of alternatives the relation between which is not determined. By invoking the importance of identifying maximal alternatives in the set of feasible allocation alternatives, we discard ineligible alternatives. In order to (2) select which alternative to choose from amongst the set of eligible alternatives, and thus to establish an actual choice, we need to invoke a different type of normative reason. Such reasons, I suggested, are created reasons that society creates through commitments generated by collective acts of willing. Commitments generated by purposeful collective acts of willing within liberal-democratic institutions provide reasons that are valid in virtue of the process through which they have been created. These reasons can guide choice in hard cases and identify which of the eligible alternatives we ought to choose.
